# Comparison of Outcomes of IVF Cycles Between Transferred Frozen Thawed Embryos and Fresh Embryos by a 2 Year Survey

**Published:** 2017

**Authors:** Ensieh Shahrokh-Tehraninejad, Zeynab Vosoog, Fatemeh Farajzadeh-Vajari

**Affiliations:** 1- Department of Endocrinology & Biomedicine Research Centre, Royan Institute for Reproductive Biomedicine, ACECR, Tehran, Iran; 2- Vali-e-Asr Reproductive & Health Research Centre, Tehran University of Medical Science, Tehran, Iran; 3- Department of Gynecology and Obstetrics, Babak Hospital, Tehran University of Medical Sciences, Tehran, Iran

**Keywords:** Ectopic pregnancy, Fresh embryo transfer, Frozen-thawed embryo transfer, In Vitro Fertilization

## Abstract

**Background:**

Infertility as one of most concerning topics in childbearing age mothers needs better managements with less complications and IVF can be assumed as an efficient method. This study aimed to compare pregnancy outcomes in fresh and frozen embryos transferred in IVF cycles.

**Methods:**

In a retrospective study, 11201 patients underwent IVF cycles from 21st March 2013 to 20th March 2014 and they were categorized into two groups according to age, previous tubal disease and surgery, tubal ligation, and previous ectopic pregnancy variables. Clinical pregnancy, ectopic pregnancy, multiple pregnancy, spontaneous abortion and preterm labor rates were compared in both groups.

**Results:**

11201 patients were categorized in two groups. Results of 4149 frozen-thawed embryo transfer cycles showed 1281 clinical pregnancies (30.9%) and 7052 fresh embryo transfer cycles which led to 2085 clinical pregnancies (29.6%) without significant differences between groups (p=0.14). Ectopic pregnancy rates in frozen and fresh groups were 38 (3%) and 52 (2.5%), respectively (p=0.409). Spontaneous abortion rates in frozen and fresh groups were 8.4% and 9.4%, respectively (p=0.32). Preterm labor was seen in both frozen and fresh groups as 28.2% and 23.4%, respectively (p=0.002) and finally, multigestational pregnancy rate was 25.7% and 22.8% in frozen and fresh groups (p=0.06).

**Conclusion:**

According to this study, frozen and fresh embryo transfer cycles were not significantly different in clinical pregnancy, ectopic pregnancy, multigestational pregnancy rates, but preterm labor was dominant in frozen group, which should not be overlooked.

## Introduction

Infertility along with social and psychological sequels concern families. They seek solutions and may finally reach assisted reproductive technology (ART). Louis Brown was born as the first successful IVF baby in 1978 that should be named delivery of new treatment of infertility (1). Such approaches yielded new unwanted results too.

Edwardo reported the first tubal ectopic pregnancy following IVF in 1976 (2) and the dilemma on effects of IVF on ectopic pregnancy appeared. It was not wise to relate these events according to available hypotheses and maybe explanations should be provided like different clinical conditions of women who underwent IVF, or various hormonal environments provided (3).

On the other hand, increased frequency of transferred frozen-thawed embryo correlates with improved pregnancy rates of frozen-thawed embryo that can be due to lower capacity of fetal compliance in endometrium under ovulation stimulation of fresh cycles, though not accepted by others (4).

Ectopic pregnancy (EP) as one of the causes of mortality in the first trimester is significant especially in childbearing period (5) and its rate is 1–2% during pregnancy and even 2.1–9.4% in artificial fertility (6, 7).

Increased ectopic pregnancy, which may be due to improved diagnostic instruments, could decline catastrophic sequels such as fertility of injured tubes and mortal bleedings by early recognition (8).

Defining methods with maximum success and minimum complications is important for mother and fetal safety and can decrease therapeutic health charges, which attracted lots of surveys containing these processes’ outcomes.

Surveys by Aflatoonian et al. (2016) illustrated results of comparison in Iranian people (9), but confined domestic data in this era obliged authors to establish a descriptive study with a big population to declare similarities and differences of pregnancy rates and unwanted results in frozen–thawed and fresh embryo transfer cycles among Iranian mothers.

## Methods

A retrospective descriptive study was established on women who had underwent cycles of embryo transfer – as blastocyst- from 21 March 2013 to 20 March 2014 at Royan Institute. The study received acceptance of ethics committee of Tehran University of Medical Sciences (TUMS). Informed consent was obtained from all individual participants included in the study. First, demographic data and history of ectopic pregnancy, tubal operations, tubal disease, tubal ligation history, type of embryo transfer cycle, and cycle outcome as pregnancy and non-pregnancy of patients were recorded.

After positive HCG test, ultrasonography was applied. Finding pregnancy sac equaled clinical pregnancy; otherwise, it was called ectopic pregnancy. Multigestational pregnancy cases were recorded as the number of fetuses, and spontaneous abortions (miscarriage) and premature deliveries.

Our inclusion criteria were women in the range of 17–43 years old and every patient whose IVF cycles. Exclusion criteria involved embryo donation or surrogate uterus and women who underwent simultaneous frozen –thawed and fresh embryo transfer and patients who had cycles due to other causes.

Captured data was analyzed in SPSS 18 software according to descriptive measures (*i.e*. frequency, mean, SD) by statistical tests like chi-square and t-test.

## Results

At the beginning, 14787 women whose IVF cycle started in mentioned period were registered. 77 cases were excluded for embryo donation or surrogate uterus. 3509 women were excluded due to other criteria, too. At last, 11201 women entered into our study and 4149 (37%) received frozen–thawed embryo transfer and 7052 (63%) were in fresh embryo transfer group.

Age averages in frozen–thawed and fresh groups were 30.78 and 32.27, respectively; however, they differed significantly (p=0.001). Data is totally illustrated in table 1, 2. There was a categorization for age as less than 35 years, between 35–39 years, and more than 39 years according to childbearing indices. Again, this categorization was not homogenous (p=0.001).

**Table 1.** Patients demographic and history data

**Frequency (%)**	**Frozen–thawed (%)**	**Fresh (%)**	**p-value**
**Age (mean**±**SD)**	30.78±5.28	32/27±5.53	0.001
Age<35	3301(39.8%)	4977(60.2%)	0.001
Age:35 39	539(31.3%)	1184(68.7%)	
Age>39	246(23.6%)	797(76.4%)	
**Abnormal tubal anatomy**	200(4.8%)	351(5%)	0.71
**previous EP**	17(0.4%)	31(0.4%)	0.81
**Tubal ligation**	0(0%)	5(0.1%)	0.16
**Previous tubal surgery**	23(0.6 %)	61(0.9%)	0.06

**Table 2.** Gathered data following embryo transfer

**Frequency (%)**	**Frozen–thawed**	**Fresh**	**p-value**
**No. Embryo transfer (mean**±**SD)**	2.83±0.738	2.30±0.823	0.001
**Clinical Pregnancy rate**	1281(30.9%)	2085(29.6%)	0.14
**Ectopic pregnancy**	38(3%)	52(2.5%)	0/409
**Spontaneous Abortion**	107(8.4%)	195(9.4%)	0/32
**Preterm labor**	3619(28.2%)	478(23.4%)	0/002
**Multigestational pregnancy**	329(25.7%)	476(22.8%)	0/06

The evaluation illustrated that 200 women (4.8%) in frozen–thawed group and 351 (5%) in fresh group had abnormal ovarian tubes which were distributed homogeneously between groups (p=0.71).

The rate of history of tubal operation in frozen–thawed and fresh group were 23 (0.6%) and 61 (0.9%), respectively (p=0.06). The number of women who had history of ectopic pregnancy in frozen–thawed and fresh groups were 17 (0.4%) versus 31 (0.4%), respectively (p=0.81).

According to recorded sheets, the number of transferred fetuses in frozen–thawed and fresh groups was 2.83 and 2.30, respectively which was significantly more in frozen transfer (p=0.001).

Clinical pregnancy rate and its relation to total embryo transfer cycles were 30.9% in frozen–thawed group and 29.6% in fresh group that did not differ significantly (p=0.14).

Ectopic pregnancy as the outcome in frozen–thawed versus fresh groups was 3% and 2.5%, respectively (p=0.41).

Spontaneous abortion rate in frozen–thawed group was 8.4% and 9.4% in fresh one (p=0.32).

Preterm labor following clinical pregnancy in frozen–thawed and fresh group were seen in 28.2% and 23.4% of subjects, respectively in which frozen–thawed group mothers were significantly more in comparison to the other group (p=0.002).

However, multigestational pregnancy was the same in groups as 25.7% in frozen–thawed and 22.8% in fresh cycles (p=0.6).

## Discussion

Frozen–thawed embryo transfer is used extensively in ART processes and involves 17% of total assisted reproductive cycles (10). Despite its encouraging properties, frozen-thawed embryo transfer may result in unwanted outcomes such as ectopic pregnancy which can change attitudes toward routinizing its application.

A recent study evaluated 11201 women who underwent embryo transfer cycles during 2 years according to unwanted outcomes in frozen–thawed and fresh cycles.

Shapiro et al. (2012) assessed 2150 blastocyst transfer cycles which illustrated more pregnancy rate in frozen–thawed group, because of hormonal stimulation as the factor reduces embryo acceptance capacity of endometrium, (11) and also, Chunjuan et al. (2014) in a survey on 1891 embryo transfer cycles showed that there was higher rate of pregnancy in frozen–thawed blastocyst compared to fresh embryo, (12) but such dominancy was not demonstrated in this study (p= 0.14).

Changli et al. (2015) recalled adnexal surgery, ectopic pregnancy history and tubal ligation as the main risk factors of ectopic pregnancy (13), while our study confirmed that these risk factors did not differ between groups and despite such homogeneity, ectopic pregnancy rates in groups were alike. Also, the previous adnexal disease or operations as risk factors for EP were not considered in this study maybe due to limited number of cases.

Extra uterine pregnancy rate was lower in frozen–thawed embryo transfer than fresh ones in a study on more than 100000 IVF cycles by Londra et al. (2015); however, such findings were not obtained in this study. The mentioned study had fabulous demographic, clinical and IVF cycle outcomes (4).

Ishihara et al. (2011) studied 10312 clinical pregnancies with frozen –thawed embryo transfer and 1352 pregnancies with fresh embryo transfer, and they assessed embryo transfer in each cycle; therefore, they omitted confounding factors (14). They gathered data from heterogeneous population and patients’ records were ignored, an issue which was included in our study.

Jun et al. (2007) evaluated the incidence of ectopic pregnancy in180 subjects of frozen–thawed group *vs.* 546 of fresh group and no significant difference was found between groups,^10^ which is in line with our data; however, tubal diseases rate was higher in freeze group which may be a confounding factor.

Our study like Ishihara did not confirm the relation between spontaneous abortions in groups (14) though multigestational pregnancy relation to embryo transfer type was not considered in this study since non-homogenous transfer in each cycle lowers its value, so preterm labor dominates in freeze cycles.

Variable volumes of groups caused heterogeneity in groups according to age and number of transferred embryos, and the lack of availability of other information like ovarian stimulation protocols and endometrium character during embryo transfer, all may affect the outcomes.

All of these shortages are limitations confined to retrospective studies that can be modulated with designing prospective studies on specific variables.

## Conclusion

Lots of studies assessed the outcome following IVF embryo transfers and in retrospective description on a large number of embryo transfers in Royan institute, there was no association among extra uterine pregnancy and embryo transfer cycles, and also clinical pregnancy or spontaneous abortion and multigestational pregnancy frequencies, but preterm labor has different scenarios and is predominant in frozen-thawed embryo transfer.
